# Insights into retinal disease and non-tubulin glutamylation from a RPGR–TTLL5 complex structure

**DOI:** 10.1083/jcb.202508020

**Published:** 2026-04-03

**Authors:** James H. Park, Richard J.Y. Liu, Xun Sun, Kishore K. Mahalingan, Suja Hiriyanna, Tiansen Li, Antonina Roll-Mecak

**Affiliations:** 1 Cell Biology and Biophysics Unit, National Institute of Neurological Disorders and Stroke, Bethesda, MD, USA; 2 Retinal Cell Biology & Degeneration Section, National Eye Institute, Bethesda, MD, USA; 3 Biochemistry and Biophysics Center, National Heart, Lung and Blood Institute, Bethesda, MD, USA

## Abstract

Mutations in retinitis pigmentosa GTPase regulator (RPGR) cause photoreceptor degeneration, vision loss, and eventual blindness. RPGR function requires glutamylation by tubulin tyrosine ligase-like 5 (TTLL5) whose mutation is also linked to severe forms of retinal degeneration. How TTLL5 targets RPGR and how mutations in either protein cause disease are unknown. Here we report the 2.8-Å X-ray crystal structure of the coactivator interacting domain (CID) of human TTLL5 in complex with the RPGR C terminus, both required for glutamylation. The RPGR C terminus forms a helix that intercalates through aromatic interactions into the CID helical bundle of novel fold. Interfacial residues are mutated in retinitis pigmentosa, as well as macular degeneration of unknown etiology. Key mutations at this interface abolish RPGR–TTLL5 interaction *in vitro* and RPGR glutamylation in mouse photoreceptors. Our work reveals mechanisms of non-tubulin substrate recognition by TTLL glutamylases, increasingly recognized as broad regulators of the proteome, and sheds light on mechanisms of disease associated with TTLL5 and RPGR mutations.

## Teaser

Park et al. present the crystal structure of an RPGR–TTLL5 co-complex, which reveals TTLL recognition paradigms for non-tubulin substrates and sheds light on disease mechanism for retinal dystrophies caused by TTLL5 and RPGR mutations.

## Introduction

**Figure 1.**
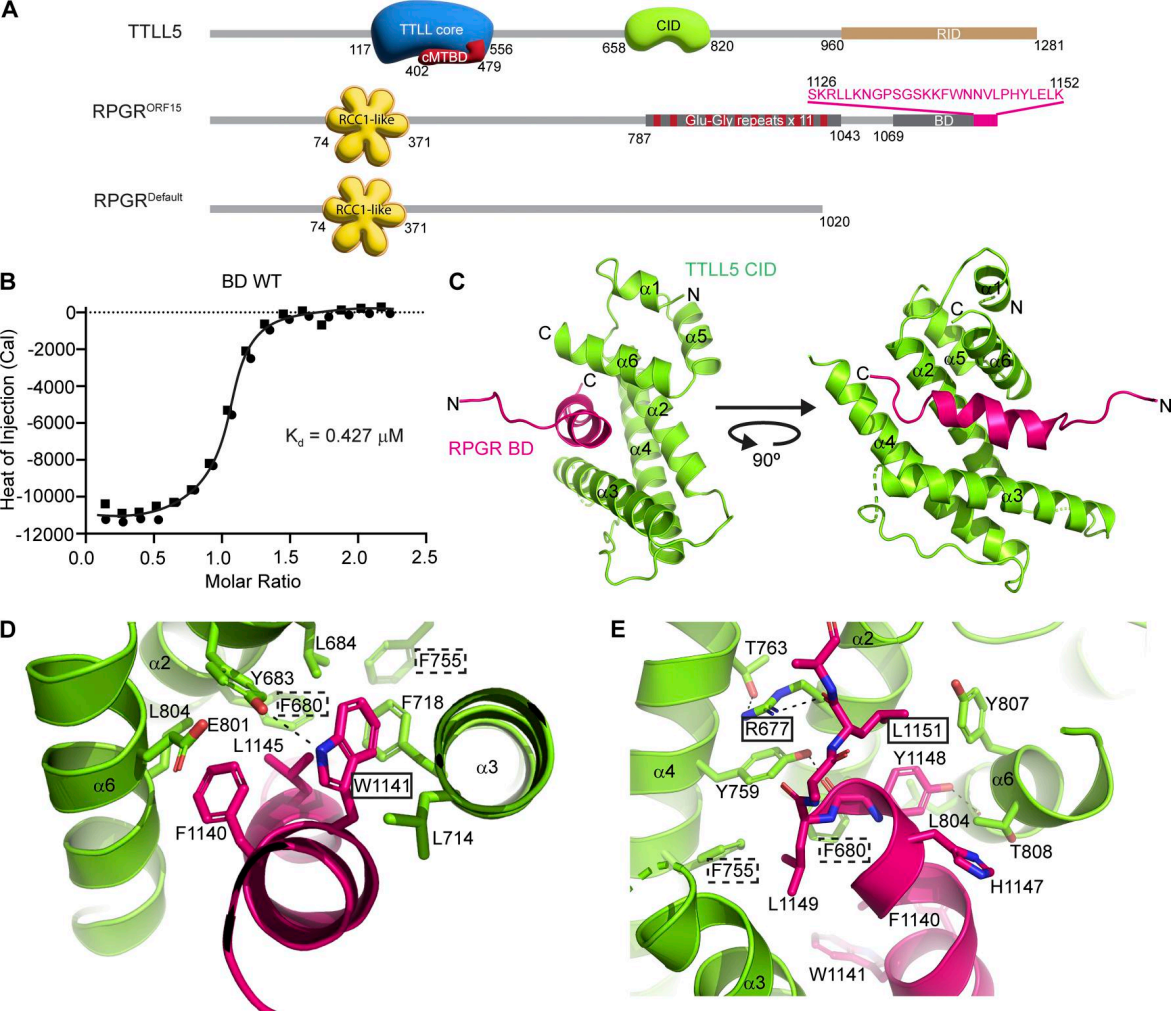
**A minimal C-terminal helix in the RPGR BD is sufficient for CID binding and intercalates into the CID helical bundle through a conserved aromatic network. (A)** Domain organization of human TTLL5 and RPGR. TTL catalytic core, blue; cMTBD, maroon; CID, green. RCC1-like β-propeller domain in RPGR, yellow, with each repeat shown as a separate propeller blade. Sequence for the minimal BD peptide required for TTLL5 targeting to RPGR shown in magenta at the top. **(B)** ITC measurements of WT miniBD peptide titrated into CID. Best fit of *n* = 2 independent experimental replicates. **(C)** 2.8-Å X-ray crystal structure of human TTLL5 CID in complex with human RPGR miniBD (Table 1) shown in cartoon representation; CID, green; RPGR, magenta. **(D and E)** Key conserved hydrophobic interactions at the CID–BD interface. Solid black boxes around residue labels designate disease-associated mutations, dashed boxes designate mutations without disease annotation in the ClinVar database that we predict are pathogenic based on our structure. RPGR and TTLL5 are colored as in B; H-bonds are shown as dashed lines.

Mutations in retinitis pigmentosa GTPase regulator (RPGR) cause 70% of X-linked retinitis pigmentosa, a degenerative retinal disease that causes blindness (Bader et al., 2003; Meindl et al., 1996; Vervoort et al., 2000). In severe cases, visual field reduction and diminished night vision manifest during childhood (Iannaccone et al., 2003). As disease progresses, photoreceptor outer segments degenerate, and retinal pigmentation diminishes. The most affected males experience total blindness by middle age (Bird, 1975). In addition to retinitis pigmentosa, RPGR mutations are found in cone-rod or cone dystrophy as well as macular dystrophy (Kolawole et al., 2023; Sergouniotis et al., 2014). Therefore, RPGR function is intimately associated with normal retinal function and aging. RPGR is a multi-domain protein with two major isoforms. The regulator of chromosome condensation 1 (RCC1)-like guanidine nucleotide exchange factor domain is present in both the default (RPGR^Default^) and retina-specific (RPGR^ORF15^) isoforms (Fig. 1 A). While disease mutations localize to the RCC1-like domain (Buraczynska et al., 1997), 80% of retinitis pigmentosa-causing mutations concentrate in the long C-terminal region of the retina-specific splice variant RPGR^ORF15^ in which a portion of intron 15 replaces exons 16–19 of RPGR^Default^ (Breuer et al., 2002; Hong et al., 2003; Mears et al., 2000; Pelletier et al., 2007; Vervoort et al., 2000) (Fig. 1 A). This extended 3′ region of ORF15 encodes Glu-Gly repeats spanning nearly 400 residues, followed by a ∼ 100 residue-long C-terminal basic domain (BD). The RPGR^ORF15^ Glu-Gly repeats are similar in length and sequence to the C-terminal tails of tubulin, which are hotspots for posttranslational regulation (Roll-Mecak, 2020). Like the tubulin tails, the RPGR^ORF15^ Glu-Gly repeats are glutamylated by a member of the tubulin tyrosine ligase-like (TTLL) family of enzymes (Sun et al., 2016). These enzymes add glutamate chains of variable lengths to internal glutamate residues in proteins (Janke et al., 2005). Glutamylation of RPGR by TTLL5 is essential for retinal health (Bedoni et al., 2016; Sun et al., 2016) and loss of TTLL5 phenocopies loss of RPGR, leading to retinal degeneration in mice (Sun et al., 2016). Consistent with roles in a shared pathway, TTLL5 mutations were found in patients diagnosed with X-linked retinitis pigmentosa as well as severe retinal degeneration (Bedoni et al., 2016; Kolawole et al., 2023; Oh et al., 2022; Sergouniotis et al., 2014).

RPGR localizes to the photoreceptor connecting cilium, a structure homologous to the primary cilium initial segment and the motile cilium transition zone. RPGR is targeted there by the photoreceptor-specific RPGR-interacting protein (RPGRIP1) (Hong et al., 2001; Shu et al., 2005) and acts as a gatekeeper of cargo trafficking into the cilia (Hong et al., 2003; Khanna, 2018). Glutamylation of RPGR by TTLL5 is necessary for cargo trafficking through the connecting cilium, including that of opsins whose failure to traffic from the cell body to the photoreceptor outer segment in RPGR and TTLL5 knockout mice is a precursor to photoreceptor degeneration (Hong et al., 2000; Sun et al., 2016).

TTLLs share a conserved catalytic domain (TTL) usually followed by a cationic microtubule-binding domain (cMTBD) (Fig. 1 A). Together, TTL and cMTBD form the minimum functional unit for microtubule binding and glutamylation (Garnham et al., 2015). Our understanding of substrate recognition by TTLL glutamylases relies exclusively on structural and mechanistic work on complexes with microtubules (Campbell et al., 2025, *Preprint*; Garnham et al., 2015; Mahalingan et al., 2024). Paradigms for non-tubulin substrate recognition for TTLL family members remain unknown even as the list of their non-tubulin substrates keeps increasing and includes, in addition to RPGR, histone chaperones Npm1 and Nap1 (Lorton et al., 2024; Miller and Heald, 2015), the PELP1 transcription factor (Kashiwaya et al., 2010), the cytosolic DNA sensor cGAS (Xia et al., 2016), and the Wnt signaling pathway protein dishevelled 3 (Kravec et al., 2024). In addition to the TTL and cMTBD domains, TTLL5 has a coactivator interaction domain (CID) and receptor interaction domain (RID) at its C terminus (Fig. 1 A). CID, originally described as a glucocorticoid receptor–interacting domain (He and Simons, 2007), binds to the BD in RPGR^ORF15^. This interaction is essential for RPGR glutamylation (Sun et al., 2016). TTLL5 does not contribute significantly to tubulin glutamylation in photoreceptors (Sun et al., 2016) but it does in primary cilia (Backer et al., 2012; He et al., 2018) and sperm tail axonemes (Bedoni et al., 2016; Lee et al., 2013; Sun et al., 2016). Consistent with this, a subset of TTLL5 mutations cause, in addition to retinal degeneration, sperm motility defects and infertility (Bedoni et al., 2016).

Understanding the RPGR–TTLL5 interaction and the mechanism of action of RPGR and TTLL5 disease variants is hampered by a lack of structural information. Because RPGR is a promising target for gene therapy (Cehajic-Kapetanovic et al., 2020; Deng et al., 2015; Gumerson et al., 2022; Hong et al., 2005; Michaelides et al., 2024; Pawlyk et al., 2016), early diagnosis of a protein defect before the onset of vision loss also has the potential to significantly improve treatment outcomes. Here we report the 2.8-Å X-ray crystal structure of human TTLL5 CID bound to a minimal RPGR^ORF15^ C-terminal helix, which we show is strictly required for recognition by TTLL5. The RPGR^ORF15^ C-terminal helix intercalates into the ⍺-helical bundle of the CID through a network of conserved aromatic residues mutated in patients with retinitis pigmentosa and macular dystrophy. Combining biochemical, cellular, and animal studies, we show that mutations of interfacial aromatic residues impair RPGR^ORF15^ glutamylation by TTLL5, with downstream defects in photoreceptor cilia. Structure-guided analysis of disease mutations enabled phenotypic classification of patient mutations, providing insights into the disease mechanism of isolated retinal phenotypes and those associated with fertility defects. Our work sheds light on molecular mechanisms underlying a major cause of vision loss and reveals for the first time the recognition mechanism between a glutamylase and its non-tubulin substrate.

## Results and discussion

### A minimal RPGR BD C-terminal helix is sufficient for TTLL5 CID recognition

To understand TTLL5 recognition of RPGR^ORF15^, we used limited proteolysis and sequence analysis to guide the design of a minimal human RPGR^ORF15^ peptide in the BD that interacts with the TTLL5 CID (Fig. 1 A, Materials and methods). Isothermal titration calorimetry (ITC) showed that this minimal RPGR^ORF15^ BD peptide consisting of residues 1,126–1,152, henceforth called miniBD, binds recombinant human CID (res. 658–820) with a *K*_d_ ∼ 0.4 μM (Fig. 1 B). The affinity of TTLL5 for miniBD is similar to that of other TTLL family glutamylases for microtubules, e.g., TTLL7 (*K*_d_ ∼ 1.9 μM) (Garnham et al., 2015) and TTLL6 (*K*_d_ ∼ 0.2 μM) (Mahalingan et al., 2024).

### X-ray structure reveals BD α-helix intercalates into the CID helical bundle

**Figure S1.**
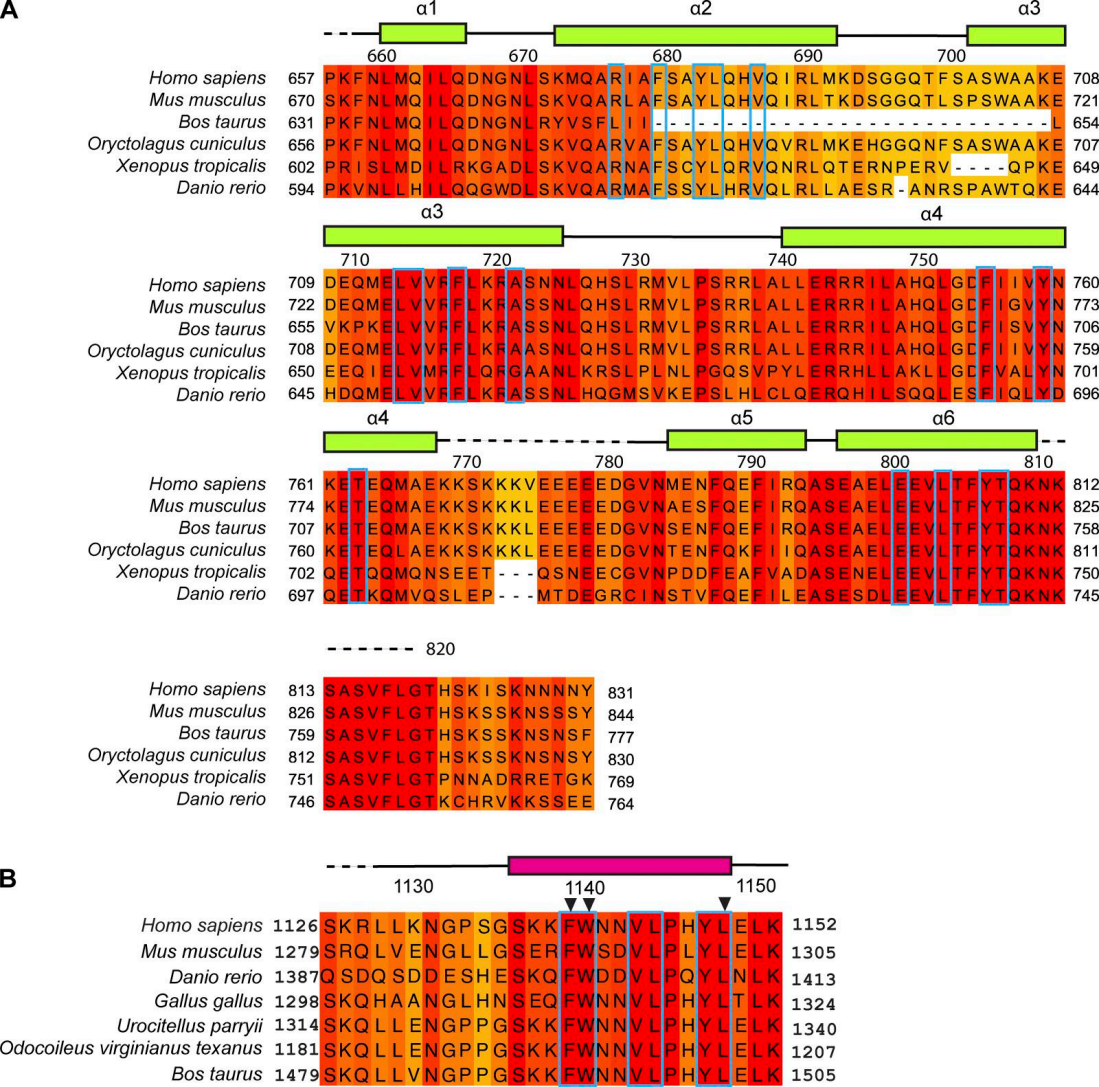
**Multiple sequence alignment of vertebrate TTLL5 CID and RPGR BD. (A)** TTLL5 CID. **(B)** RPGR miniBD. Secondary structure elements indicated above the sequence and based on our X-ray crystal structure of the complex; gaps of disordered, unmodeled residues are indicated by a dotted line. Hydrophobic residues at the CID–BD interface indicated with blue boxes. Residues mutated for ITC experiments indicated with black arrows above the sequence.

**Figure S2.**
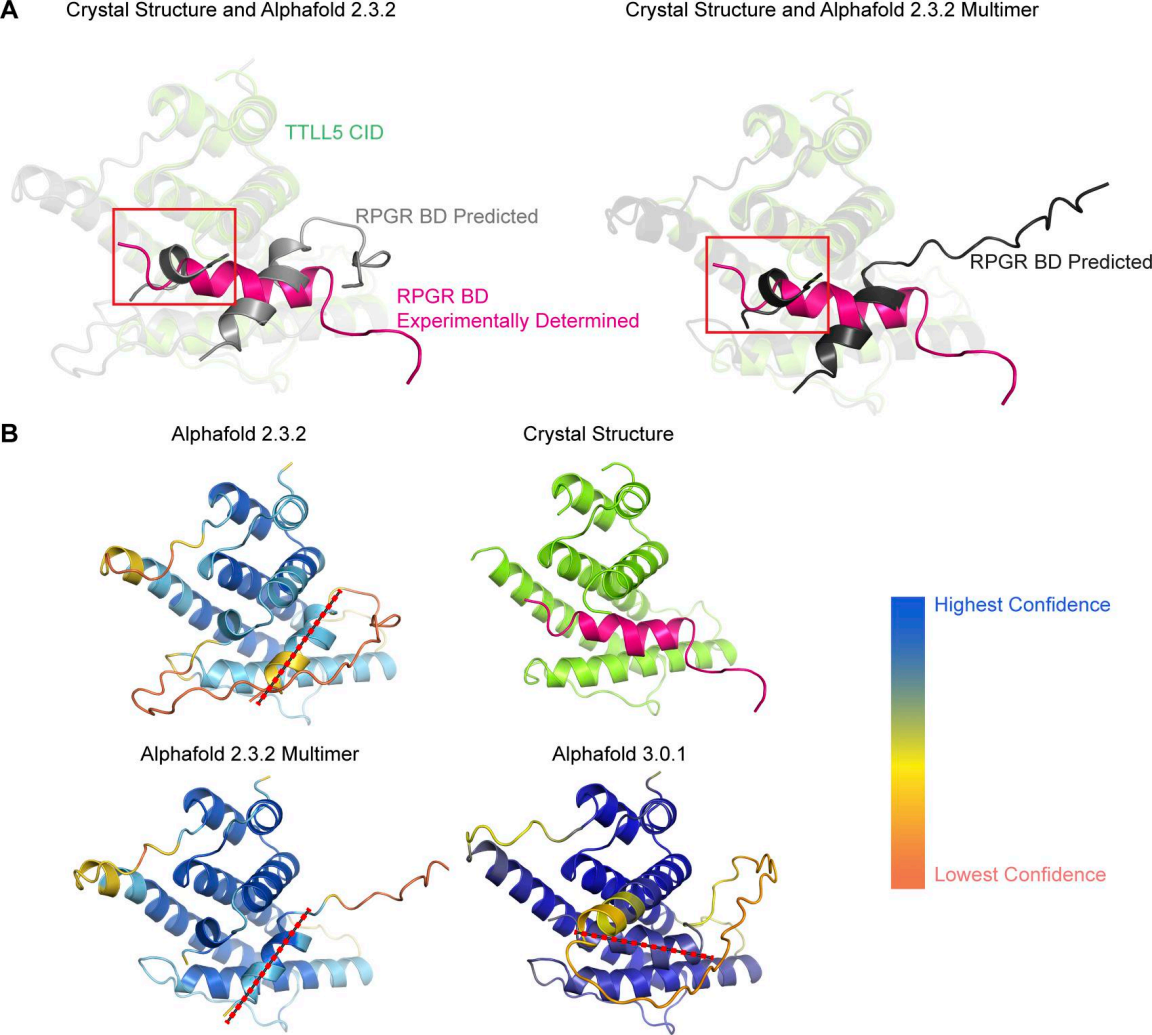
**Comparison of the experimental CID-miniBD X-ray crystal structure with AlphaFold predictions. (A)** Top, AlphaFold 2.3.2 predicted structures of fused CID–BD (light gray), similar to the construct we crystallized, and AlphaFold 2.3.2 multimer prediction of the complex with CID and BD as separate chains (dark gray) superimposed on the X-ray crystal structure of the CID–BD complex with chains colored as in Fig. 1. TTLL5 CID 658–811 rendered transparent; residues 812–820 in TTLL5 CID are highlighted by a red box. Our analysis suggests that the incorrect modeling of these residues by AlphaFold result in an incorrect prediction of the position of the BD helix in the CID–BD complex. **(B)** X-ray crystal structure of the CID–miniBD complex compared with AlphaFold 2.3.2, AlphaFold 2.3.2 multimer, and AlphaFold 3.0.1 predictions. Dotted red line overlaying predicted structures indicates orientation of miniBD. Alphafold prediction structures colored by confidence score. Both AlphaFold 2.3.2 and AlphaFold 2.3.2 multimer assign medium to high confidence to the incorrectly predicted BD helix, with high confidence for the entire miniBD helix assigned by AlphaFold 2.3.2 multimer.

To elucidate the mechanism of RPGR recognition by TTLL5, we determined the X-ray crystal structure of human CID (res. 658–820) in complex with miniBD (*R*_free_ = 0.289, *R*_work_ = 0.258; Materials and methods, Table 1). The structure revealed that the CID consists of a bundle of six ⍺-helices (Fig. 1 C). Search of the PDB using the Dali server (Holm et al., 2023) revealed no homologous structures. The top hit (*Megavirus chilensis* nucleotidyl transferase, PDB ID 4AMS) had a Z-score of 5.5 and structural homology only with helices α2, α3, and α4 of CID. Similarly, Foldseek (van Kempen et al., 2024) did not identify any structural homologs. Thus, TTLL5 CID has a previously unreported fold. The RPGR^ORF15^ miniBD folds into a helix (res. 1,136–1,149), which is recognized in a hydrophobic groove formed by helices α2, α3, and α6 of the CID (Fig. 1 C). The CID-miniBD interface is conserved across vertebrates (Fig. S1), indicating a shared binding mode. We note that while AlphaFold (Jumper et al., 2021) predictions for the isolated CID and miniBD domains were correct, the model for the complex was not, with the BD helix rotated ∼ 30° relative to the real orientation, which, unlike in the prediction, provides optimal packing in the hydrophobic groove of the CID (Fig. S2 A). The incorrect location of the BD helix was predicted with high confidence by AlphaFold multimer (Evans et al., 2022, *Preprint*), but this helix was eventually correctly positioned with AlphaFold 3 (Abramson et al., 2024) (Fig. S2 B), although we note that we shared our structure with the AlphaFold team by that time.

**Table 1.** X-ray data collection and refinement statistics

​	CID_BD PDB: 9PHH
**Data collection**	
Space group	P 41 21 2
Wavelength	0.97741
Beamline	ALS 501
***Cell dimensions***	
*a*, *b*, *c* (Å)	62.9, 62.9, 166.6
α, β, γ (°)	90, 90, 90
Resolution (Å)*	44.48–2.80 (8.85–2.80)
*R*_merge*_	0.060 (0.428)
*I*/σI*	36.0 (7.9)
Completeness (%)*	100 (100)
Redundancy*	25.1 (25.3)
**Refinement**	
Resolution (Å)*	42.98–2.80 (2.98–2.80)
No. reflections*	8,823 (1,422)
*R*_work_/*R*_free*_	0.258/0.289 (0.428/0.459)
***No. atoms***	
Protein	1,223
Ligand/ion	0
Water	0
***B-factors***	​
Protein	60.3
Ligand/ion	–
Water	–
MolProbity	1.27 (100th percentile)
***R.m.s. deviations***	
Bond lengths (Å)	0.24
Bond angles (°)	0.41
***Ramachandran***	
Favored (%)	149 (94.3)
Allowed (%)	9 (5.7)
Outlier (%)	0 (0)

* Values in parentheses are for highest-resolution shell.

The CID–BD interface is rich in conserved aromatic residues. At the center of the interface, invariant RPGR F1140, W1141, and L1145 are embedded in a large hydrophobic socket in the TTLL5 CID, formed by conserved residues F680, Y683, and L684 in helix α2, invariant residues L714 and F718 in α3, and invariant residues E801 and L804 in α6. These van der Waals interactions are augmented by hydrogen bonds between Y683 and the indole ring of W1141 in RPGR (Fig. 1 D). At the C terminus of the miniBD helix, invariant Y1148 in RPGR rests in a smaller hydrophobic socket formed by invariant TTLL5 residues L804, Y807, and T808 in α6 and hydrogen bonds through its hydroxyl group to the backbone carbonyl of L804 in TTLL5 (Fig. 1 E). Invariant L1149 in RPGR forms a hydrophobic endcap against TTLL5 F680 and invariant F755. Interaction with the miniBD C terminus is mediated through hydrogen bonds between the backbone carbonyl of RPGR Y1148 and the hydroxyl group of invariant TTLL5 Y759 and between the backbone carbonyl of RPGR L1151 and the guanidinium group of conserved TTLL5 R677. The latter is positioned at this interface by van der Waals interactions with the aromatic ring of Y759 and a H-bond with invariant T763 in TTLL5 (Fig. 1 E).

Several retinal disease-associated mutations are at the CID–BD interface. RPGR W1141C (ClinVar VCV001012373.2) removes a major hydrophobic interaction at this interface and weakens the complex. The L1151F (Clinvar VCV000438145.2) mutation results in a clash with TTLL5 Y807 and the N terminus of ⍺2 (Fig. 1 D), disrupting this interface. TTLL5 R677Q (Clinvar VCV000867152.9) weakens the CID–BD interface through the loss of a H-bond. Moreover, our structure reveals that several TTLL5 mutations with no disease annotations in the ClinVar database may be pathogenic: F680L (ClinVar VCV001494309.3) destabilizes the hydrophobic core; F755C (Clinvar VCV001483516.5) and F755L (Clinvar VCV001004837.7) both weaken the interface with RPGR by reducing van der Waals interactions with L1149. Thus, our structure reveals that disruption of the CID–BD interface, either through RPGR or TTLL5 mutations is associated with retinal disease, consistent with the importance of targeting TTLL5 to RPGR for normal function.

### CID–BD interface mutations impair RPGR glutamylation in photoreceptors

**Figure 2.**
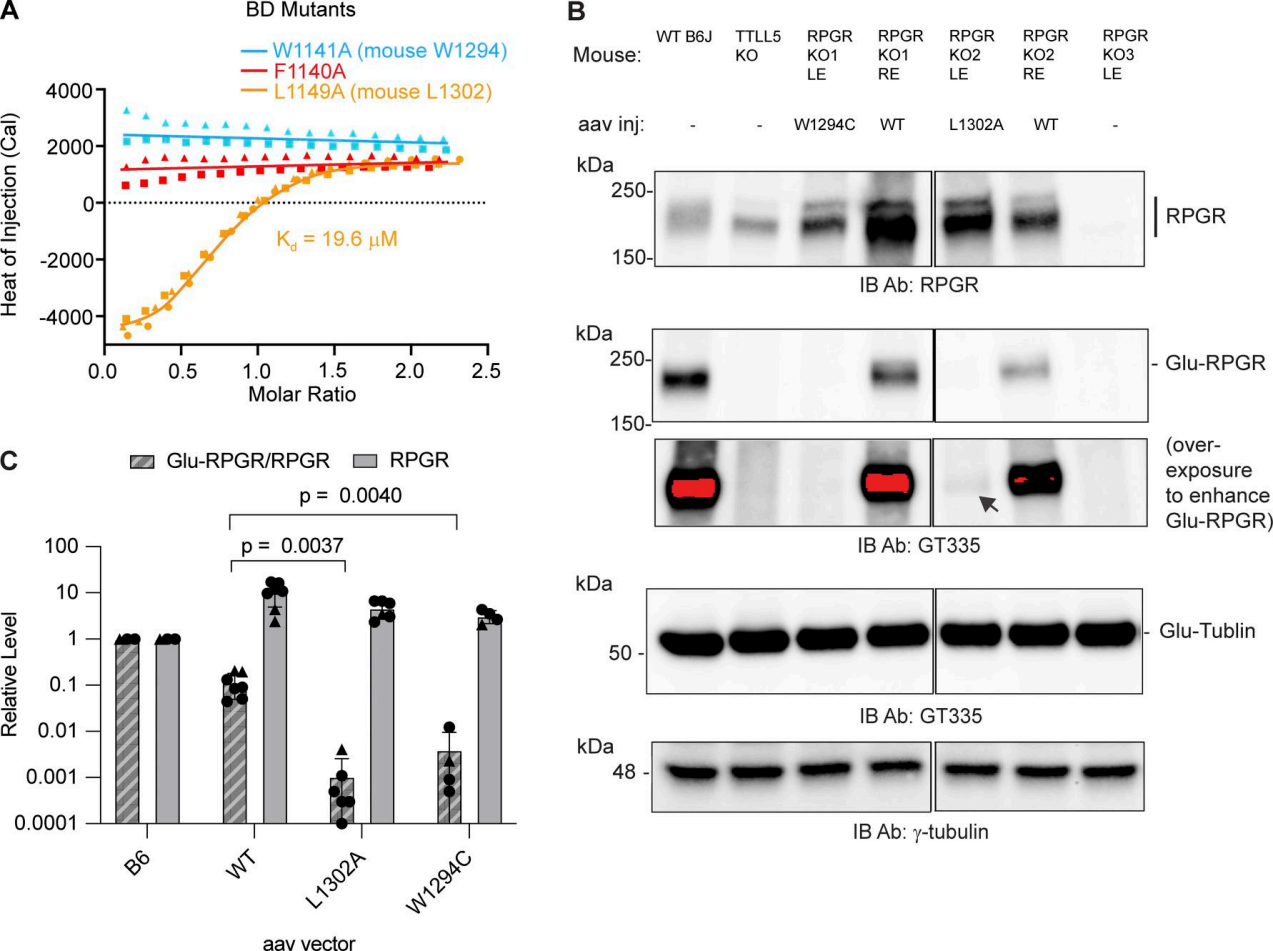
**The aromatic network at the CID–BD interface is essential for complex formation and RPGR glutamylation. (A)** ITC measurement of F1140A, W1141A, and L1149A mutant miniBD peptides titrated into WT CID; *n* = 2 independent experimental replicates for both W1141A and F1140A mutant peptides, shown as triangles and squares; *n* = 3 independent experimental replicates for L1149A, shown as triangles, squares and circles. **(B)** Western blot of RPGR expression and glutamylation in one set of RPGR knockout mice injected with WT or mutant RPGR. Black arrow in overexposed RPGR blot indicates trace glutamylation of RPGR L1302A. Two WT mice are shown to illustrate the variability of replicate injections. Saturated pixels appear in red. Double dotted lines indicate cropping to exclude samples with failed AAV injection (uncropped Western blots, including samples with failed AAV injection provided as Source Data) LE, left eye; RE, right eye. **(C)** RPGR glutamylation signal normalized to RPGR expression (Glu-RPGR/RPGR, hashed bars) and RPGR levels (solid bars) for WT and mutants in AAV-injected RPGR KO mice. The RPGR expression levels are similar between the WT and mutants and higher than endogenous. Each circle or triangle indicates one retina analyzed. Circles and triangles denote different secondary antibody detection scheme (Materials and methods). Uncropped western blots including samples with failed AAV injection are provided in Source Data. Bars indicate mean ± SD. *n* = 7 biological replicates for RPGR WT, six for RPGR L1302A, and four for RPGR W1294C. P values from two-tailed unpaired Welch’s *t* test. Source data are available for this figure: SourceData F2. KO, knockout.

To investigate the contribution of key residues at the CID–BD interface, we synthesized three miniBD peptides corresponding to human RPGR^ORF15^ mutants F1140A, W1141A, and L1149A (Fig. S1, Materials and methods). F1140 and W1141 anchor the center of the miniBD helix within the CID bundle, while L1149 forms a stabilizing cap at the C terminus of the miniBD helix (Fig. 1, D and E). Consistent with our structure, we observed no measurable binding between TTLL5 and F1140A and W1141A RPGR mutant peptides (Fig. 2 A). The L1149A mutant peptide has a ∼ 50-fold reduction in binding affinity (19.6 μM versus 0.4 μM for mutant and WT, respectively), congruent with the more peripheral location of L1149 relative to the CID hydrophobic socket (Figs. 1 E and 2 A).

We next examined the effect of our structure-guided RPGR point mutations in photoreceptors. For this, we used adeno-associated virus (AAV) to deliver WT or mutant mouse RPGR into the retina of RPGR knockout mice (Hong et al., 2005). A similar protocol rescues RPGR expression and prevents photoreceptor degeneration in this mouse line (Pawlyk et al., 2016). We cloned the mouse equivalents of human RPGR W1141C (mouse RPGR W1294C) and L1149A (mouse RPGR L1302A). The former is of particular interest not only because it disrupts a key residue at the CID–BD-binding interface but also because this mutation is found in a patient with macular dystrophy (Clinvar VCV001012373.2). Because macular dystrophy is also a retinal degeneration disease, the RPGR W1141C mutation is likely causal. AAV injection led to high expression of both WT and mutant RPGR compared with the endogenous protein (Fig. 2, B and C). Only a fraction of the overexpressed WT RPGR is glutamylated by TTLL5, likely reflecting that not all overexpressed RPGR targets to the connecting cilium (Hong et al., 2005). Consistent with our structural analysis, both W1294C and L1302A RPGR mutants are markedly less glutamylated compared with the injected WT RPGR (Fig. 2 B). Quantification from multiple injected retinas shows RPGR W1294C glutamylation is decreased >10-fold compared with WT, while the L1302A mutant is decreased ∼ 100-fold (Fig. 2 C). These *in vivo* results are consistent with our solution biophysics data that show large impairment in binding to the TTLL5 CID for these two mutations (Fig. 2 A). Both mutants have similar tubulin glutamylation levels (Fig. 2 B), indicating that the integrity of the CID–BD interface is important for targeting TTLL5 to RPGR, but not to tubulin or microtubules. Taken together, our results show that the CID–BD interface is necessary for RPGR glutamylation in photoreceptors and reveal that a RPGR mutation found in macular dystrophy results in complete loss of RPGR glutamylation *in vivo*, implicating glutamylation by TTLL5 in this retinal disease also.

**Figure 3.**
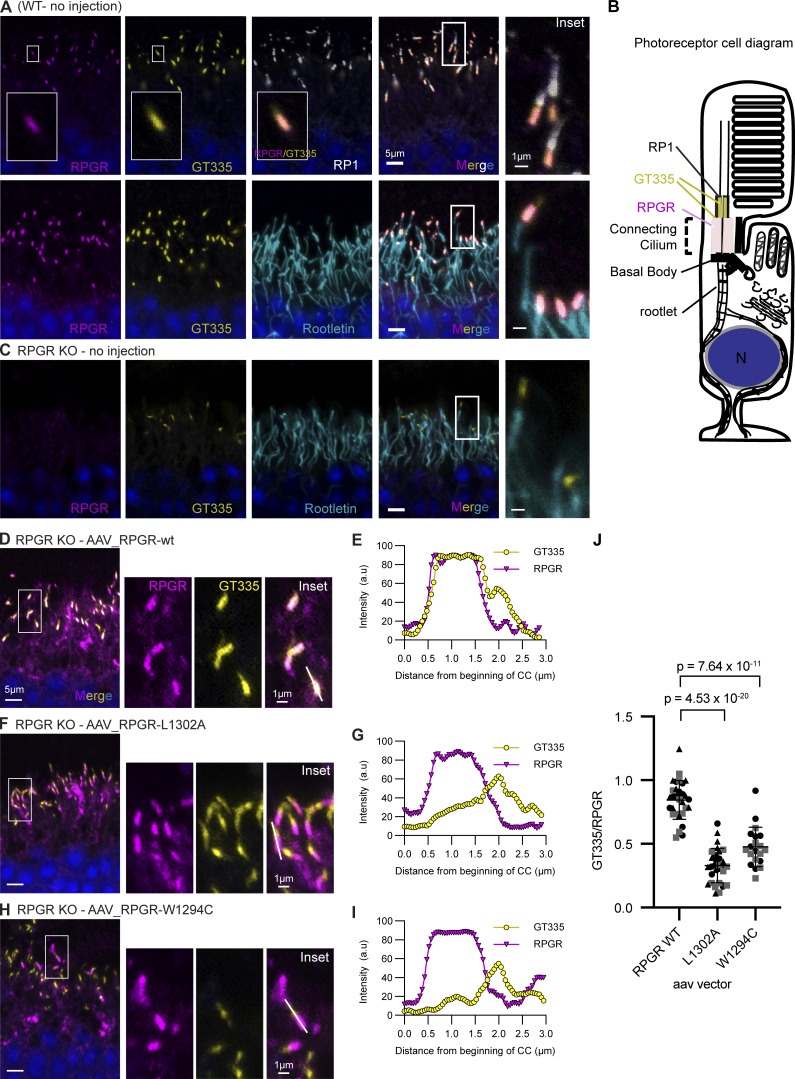
**RPGR glutamylation at the connecting cilium is mediated by the CID–BD interaction. (A)** Ciliary region of a WT mouse retina stained for RPGR (magenta), GT335 (yellow), RP1 (white; upper panel), and rootletin (cyan; lower panel). White boxes in the RPGR and GT335 channels show a magnified single connecting cilium. Smaller white box in the merged channels panel indicates magnified region shown in inset panel on the right. RPGR localizes to the connecting cilium, equivalent to the transition zone in the primary cilium (for photoreceptor diagram see B). GT335 also stains the transition zone and extends distally beyond the connecting cilium in a domain that overlaps with the photoreceptor protein RP1. The ciliary rootlet, stained by the rootletin antibody, extends proximally away from the connecting cilium. RPGR signal fully overlaps with GT335 signal in the connecting cilium (pink, inset), and glutamylated RPGR accounts entirely for the GT335 signal within the connecting cilium (Sun et al., 2016). **(B)** Schematic diagram of a photoreceptor cell, highlighting the ciliary region, with different domains stained by antibody markers indicated. **(C)** The ciliary region of an RPGR knockout (KO) mouse retina stained with RPGR (magenta), GT335 (yellow), and rootletin (cyan). Both RPGR and GT335 signals are absent from the connecting cilium, and GT335 stains only a domain distal from the connecting cilium, likely containing glutamylated tubulin. The connecting cilium appears as a gap between the rootletin and GT335 signals (inset). **(D)** Representative image of the ciliary region of a RPGR knockout (KO) mouse injected subretinally with an AAV vector carrying WT RPGR. The staining pattern largely resembles that of the WT mouse retina, where RPGR and GT335 colocalize to the connecting cilium and appear pink on the merged image (inset). The injected retina shows some background because recombinant RPGR does not cleanly localize to the connecting cilium, as documented previously (Hong et al., 2005). Importantly, only RPGR localized to the connecting cilium is glutamylated, as evidenced by the GT335 signal. **(E)** Line trace of GT335 (yellow) and RPGR (magenta) signal along the connecting cilium marked with a white line in the inset of the overlay in D. **(F)** Representative image of matching ciliary region of an RPGR knockout (KO) mouse injected subretinally with an AAV vector carrying the L1302A mutant form of RPGR. The L1302A mutant localizes to the connecting cilium, like WT RPGR, but is not glutamylated as evidenced by the lack of GT335 signal (inset). **(G)** Line trace as in E. **(H)** Representative image of matching ciliary region of an RPGR knockout (KO) mouse injected subretinally with an AAV vector carrying the W1294C (human equivalent W1141C) disease mutant form of RPGR. As with L1302A, there is no co-staining of mutant RPGR with GT335 (inset), indicating lack of glutamylation. **(I)** Line trace as in E. Only WT RPGR injections (E) show correlated GT335 and RPGR signal. **(J)** Ratio of GT335:RPGR signal at point of maximum RPGR signal obtained from line traces of multiple connecting cilia for WT and RPGR mutants. Each data point indicates an individual cilium analyzed. Bars indicate mean ± SD. P values from two-tailed unpaired *t* test. *n* = 30 cilia for WT (three injections), *n* = 30 cilia for L1302A (three injections), and *n* = 20 cilia for W1294C (two injections). Data points originating from separate injections are indicated by squares, circles, and triangles. For additional examples of intensity profiles see Fig. S3.

**Figure S3.**
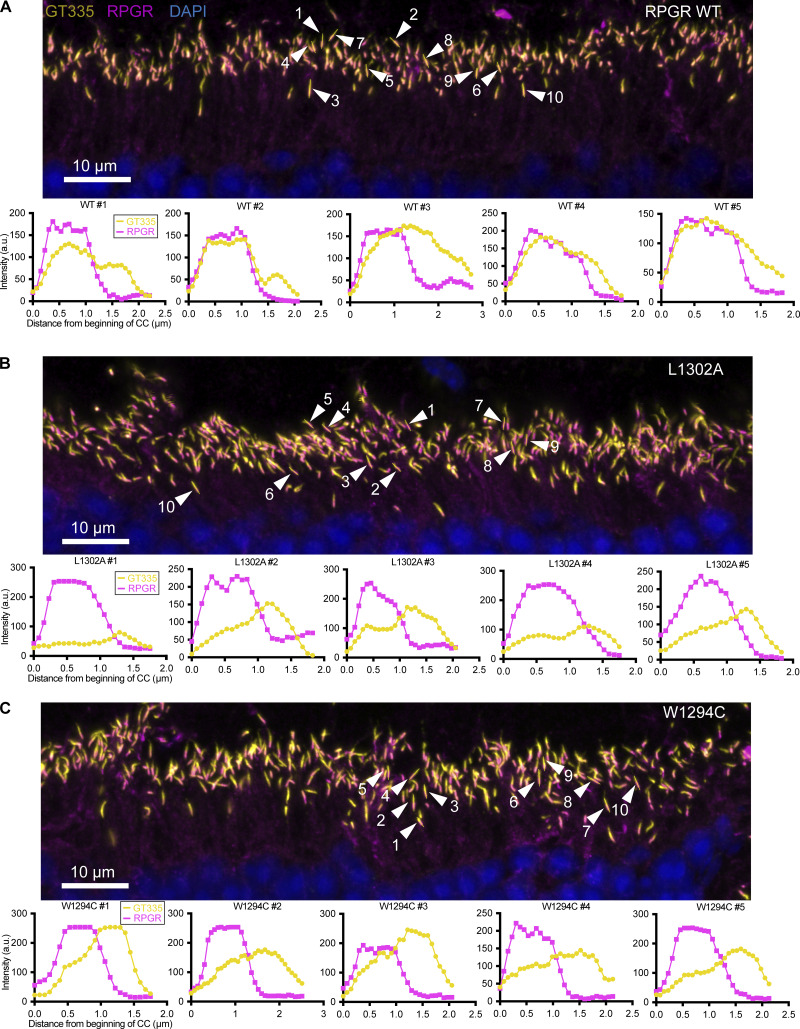
**Traced cilia and representative intensity profiles from one biological replicate quantified in Fig. 3 J. (A–C)** WT, (B) L1302A, and (C) W1294C RPGR AAV-injected mouse retina. Top for each condition: micrographs of immunostained mouse retina with GT335 in yellow, RPGR in magenta, and DAPI in blue. White arrows point to each of 10 traced connecting cilia, with yellow lines indicating traces. Cilia were manually traced and intensities extracted in ImageJ. Bottom for each condition: five intensity profiles of GT335 (yellow) and RPGR (magenta) along line traces, numbered according to panel above. Mice injected with WT RPGR AAV have strong GT335 signal at the proximal portion of their connecting cilia where RPGR is localized, while both mutant forms of RPGR show low GT335 signal where RPGR is localized.

The photoreceptor connecting cilium where RPGR localizes divides the cell body compartment, which contains rootletin, and the photoreceptor outer segment, which contains retinitis pigmentosa 1 (RP1). To assess whether the defects in RPGR glutamylation are not due to failure of the mutants to localize correctly, we visualized RPGR by immunofluorescence microscopy using either rootletin or RP1 as markers (Sun et al., 2016). As expected, immunofluorescence of WT mouse retina shows overlapping RPGR and glutamylation staining (detected by GT335) adjacent to rootletin and RP1 staining (Fig. 3 A). The RPGR KO mouse has no RPGR or glutamylation signal in this region, while the outer segment shows no changes in glutamylation between WT and RPGR KO (Fig. 3 C), in line with previous observations that RPGR^ORF15^ and not tubulin is the major glutamylated molecule in the connecting cilium (Sun et al., 2016). Staining of retina from AAV rescue mice reveals that WT RPGR as well as the two interface mutants, W1294C and L1302A, localize to the connecting cilium (Fig. 3, D–I). However, only WT RPGR restored glutamylation in this compartment (Fig. 3 D). Analysis of multiple cilia shows that, unlike for the WT, where the GT335 and the RPGR signals overlap closely (Fig. 3 E and Fig. S3 A), glutamylation does not overlap with RPGR staining for the W1294C and L1302A mutants (Fig. 3, F–I; and Fig. S3, B and C). This confirms that these mutations prevent RPGR glutamylation and that the observed loss in glutamylation is not due to failure to target to the connecting cilium, consistent with earlier reports that RPGR localization is not affected by TTLL5 KO (Pawlyk et al., 2016; Sun et al., 2016). To quantitate the difference in glutamylation, we traced connecting cilia from WT and each mutant RPGR AAV rescue and measured the fluorescence intensity of GT335 and RPGR staining. In cilia expressing WT RPGR, the maxima GT335 and RPGR signals overlap closely (Fig. 3 E and Fig. S3 A). In contrast, GT335 signal is weaker and poorly correlated with that for the L1302A (Fig. 3G and Fig. S3 B) and W1294C RPGR mutants (Fig. 3 I and Fig. S3 C), indicating impaired RPGR glutamylation. Quantitation of GT335 signal normalized to RPGR at the point of maximum RPGR signal shows that this ratio is significantly higher in the WT compared with the L1302A and W1294C mutants (Fig. 3 J), confirming the impaired glutamylation of these mutants. *In toto*, our results indicate that RPGR localization is independent of glutamylation and that the conserved aromatic residues at the CID–BD interface are necessary for RPGR glutamylation but not localization.

RPGR^ORF15^ requires glutamylation for its function in photoreceptors where it regulates opsin trafficking (Cehajic-Kapetanovic et al., 2022; Hong et al., 2005; Pawlyk et al., 2016; Sun et al., 2016). The high degree of conservation across vertebrates of the aromatic-rich sequence in the RPGR^ORF15^ C terminus was noted when this isoform was initially identified (Vervoort et al., 2000). Now our 2.8-Å resolution X-ray crystal structure of a CID–BD complex (Fig. 1) reveals that this aromatic-rich sequence forms a short helix that nestles within the α-helical bundle of the CID and is responsible for targeting TTLL5 to RPGR to glutamylate it. By combining biophysical assays with localized genetic rescue of RPGR KO mice, we show that mutations that disrupt the CID–BD interface impair RPGR glutamylation, which is needed for photoreceptor maintenance. Thus, the CID is an RPGR-specific adapter in TTLL5.

**Figure 4.**
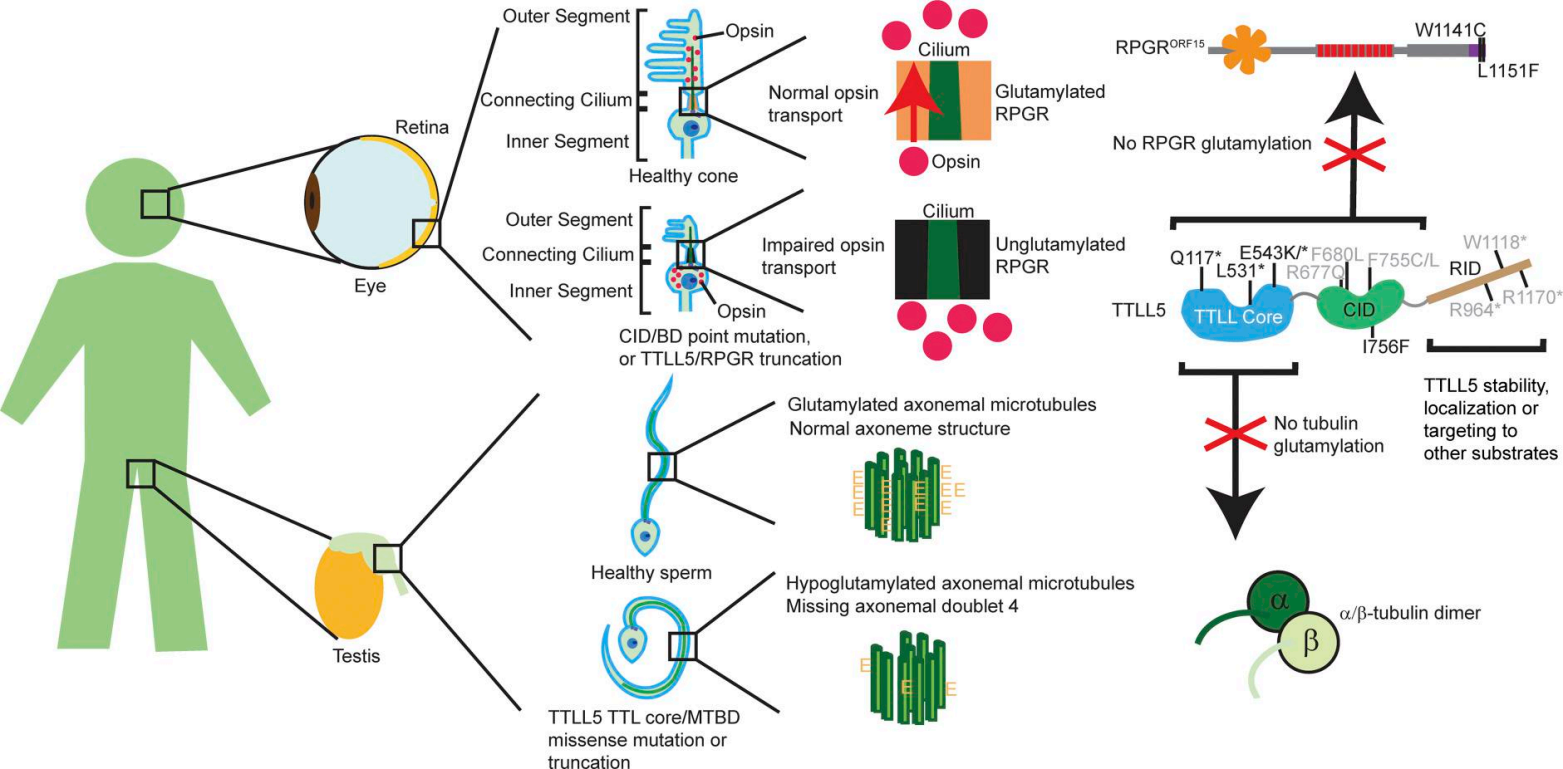
**Proposed model for the division of substrate recognition between the TTLL5 core and CID leading to distinct disease manifestations.** Schematic showing how RPGR and TTLL5 mutations impact distinct specialized cells in retina and male reproductive system (left). In healthy photoreceptors, opsin (red dots) is trafficked (red arrow) through the connecting cilium containing glutamylated RPGR to the outer segment. In RPGR/TTLL5 mutations, opsin trafficking is disrupted, leading to outer segment degeneration (middle, top). In healthy sperm, TTLL5 glutamylates tubulin in axonemes (axonemal doublets in dark and light green). When TTLL5 recognition of tubulin is disrupted, axonemal glutamylation is insufficient, and sperm morphology and motility are compromised (middle, bottom). Right: at the protein level, our results suggest that mutations in RPGR miniBD region and point mutations in TTLL5 CID specifically impact RPGR glutamylation in photoreceptors but not tubulin glutamylation in sperm axoneme. Mutations in TTLL5 catalytic domain or MTBD cause loss of both RPGR and tubulin glutamylation. Additionally, mutations in TTLL5 RID may cause retinal degeneration by impacting protein stability, localization, or recognition of non-TTLL5 and non-RPGR substrates. TTLL5 and RPGR domains colored according to Fig. 1 A. Disease-associated RPGR^ORF15^ and TTLL5 mutations reported previously (Bedoni et al., 2016) are indicated in black on the protein schematic. Additional mutations from the ClinVar database that we predict cause retina-specific disease are shown in gray.

Our work shows how the modular architecture of TTLL5 enables it to modify both microtubules and RPGR through its adapter domains: the cMTBD for microtubules and the CID for RPGR. These distinct targeting modules provide an explanation for the disparate disease outcomes in patients with different TTLL5 mutations. For example, TTLL5 I756F, a point mutation in CID helix ⍺4, causes retinal degeneration but not male infertility (Bedoni et al., 2016). I756 lies at the interface between α2 and α4 of the CID. Its substitution to a bulky phenylalanine destabilizes these helices and likely impairs BD binding and RPGR glutamylation. The cMTBD is unaffected, likely leading to normal microtubule recognition and glutamylation. In contrast, truncations and point mutations in the catalytic domain of TTLL5 or cMTBD, which folds against the catalytic domain (Garnham et al., 2015; Mahalingan et al., 2024), cause not only retinal degeneration but also sperm motility defects (Fig. 4) (Bedoni et al., 2016; Lee et al., 2013) and, in some cases, hearing loss (Oh et al., 2022) consistent with defects both in glutamylating RPGR in the retina and tubulin in the axoneme. Two disease-associated point mutations were identified in the TTLL5 cMTBD: R409Q (Clinvar VCV000851210.8) in a patient with cone-rod dystrophy and R478Q in a female patient with severe retinal degeneration (Kolawole et al., 2023). In the TTLL structures known to date, TTLL6 (Mahalingan et al., 2024), TTLL7 (Garnham et al., 2015), and TTLL11 (Campbell et al., 2025, *Preprint*), the cMTBD packs against the TTL-like core, and therefore these mutations likely impact not only microtubule binding but also catalytic activity. Interestingly, mutations in the RID, including R964* (Clinvar VCV002888538.7) and W1118* (Clinvar VCV000139515.3), were found in patients with cone-rod dystrophy 19, while R1170* (Clinvar VCV000866515.8) was found in a patient with retinal dystrophy, suggesting that the RID is also functionally relevant in photoreceptors (Fig. 4). Using TTLL5 and RPGR overexpression in HEK293 cells, we previously observed that the TTLL5 W1118* mutant glutamylates tubulin and RPGR but not higher molecular weight proteins endogenous to the HEK293 that the WT modifies, suggesting that the RID may target TTLL5 to other substrates (Sun et al., 2016). Alternatively, the RID mutations may affect TTLL5 expression or stability in photoreceptors. The molecular mechanisms of disease mutations in this region of TTLL5 will require future studies.

TTLL glutamylases add glutamate chains of variable lengths to proteins by first adding a branched glutamate on an internal glutamate in the target protein—initiation—followed by the subsequent elongation of this branch (Mahalingan et al., 2020; van Dijk et al., 2007). The active site signature of TTLL5 is consistent with it functioning as a glutamyl initiase (Mahalingan et al., 2020), i.e., it adds primarily monoglutamates to substrates. This activity is consistent with immunoblotting of RPGR from photoreceptors, which revealed that RPGR is mostly monoglutamylated, with low signal for longer polyglutamate chains (Sun et al., 2016). Interestingly, hyperglutamylation due to defects in cytosolic carboxypeptidase 5 (CCP5), which strictly removes monoglutamates (and not polyglutamate chains) from tubulin and non-tubulin substrates (Chen et al., 2024), causes retinal degeneration (Aljammal et al., 2024; Mercey et al., 2024). These results suggest that homeostasis of monoglutamates on RPGR achieved through the balance of TTLL5 and CCP5 activity is important for photoreceptor maintenance and function. Tubulin glutamylation regulates the binding and activity of microtubule-associated proteins tau (Boucher et al., 1994; Genova et al., 2023), MAP1A and MAP1B (Bonnet et al., 2001), kinesin motors (Lessard et al., 2019; Sirajuddin et al., 2014), and microtubule-severing enzymes spastin (Lacroix et al., 2010; Ten Martin et al., 2025; Valenstein and Roll-Mecak, 2016) and katanin (Sharma et al., 2007; Szczesna et al., 2022; Ten Martin et al., 2025; Zehr et al., 2020). RPGR glutamylation could similarly modulate the recruitment of effectors to the connecting cilium, where they could function as part of a barrier that selectively allows transport into the photoreceptor outer segment.

In addition to offering insight into the mechanism of TTLL5 targeting to RPGR and the etiology of retinal diseases caused by TTLL5 and RPGR mutations, our work also provides the first mechanistic insight into the targeting of a TTLL family enzyme to a non-tubulin substrate. Biochemical and cellular overexpression studies indicate that TTLL5 and TTLL4 have multiple non-tubulin substrates (van Dijk et al., 2008). Therefore, specialized recognition domains like the CID may be a more general strategy for non-tubulin substrate recognition by TTLLs. Future studies will be needed to dissect the complex regulatory interactions that target TTLL glutamylases to their diverse cell type– and tissue-specific substrates.

## Materials and methods

### Protein expression and purification

The mEGFP-SUMO-CID (human res. 658–820) -BD (human res. 1,126–1,152) fusion cassette was synthesized and cloned into the pET28a backbone using NheI and EcoRI restriction sites. To express human TTLL5 CID (res. 658–820) for ITC measurements, a STOP codon was inserted by site-directed mutagenesis into the linker between the Human TTLL5 658–820 (NP_055887.3) and Human RPGR 1126–1,152 (NP_001030025.1) in the vector used to express CID–BD. Vectors were sequenced by Psomagen standard Sanger sequencing service.

CID–BD, 6xHis-mEGFP-SUMO-TTLL5 (res. 658–820)-RPGR (res. 1,126–1,152), and TTLL5 CID, 6xHis-mEGFP-SUMO-TTLL5 (res. 658–820) were expressed in LOBSTR *Escherichia coli* (Andersen et al., 2013). Bacteria was grown in 4-L conical flasks containing 2 L PowerBroth media (AthenaES 0106) with 50 μg/ml kanamycin at 37°C with 210 rpm orbital agitation to OD_600_ of 1.2–1.8 and induced with 1 mM IPTG at 16°C overnight. Cells were harvested by centrifugation using a JLA 8.1,000 rotor (363688; Beckman Coulter) at 4,500 rpm for 15 min. Media was removed, and cell pellet was resuspended in lysis buffer (50 mM Tris, pH 8, 300 mM NaCl, 5 mM MgCl_2_, 2 mM CaCl_2_, and 10% glycerol) with 0.5 mM TCEP, 0.17 mg/ml PMSF, 1 μg/ml leupeptin, 2 μg/ml aprotinin, and 1 μg/ml pepstatin added at the time of use. 50 ml lysis buffer was used per 4 L culture. Resuspended cells were either lysed immediately for protein purification or flash frozen in liquid nitrogen and stored at −80°C until needed.

Frozen cells were partially thawed in a water bath kept at 37°C and then placed on ice to fully thaw. Cells were lysed by passing three times through a microfluidizer. Lysate was digested with 1:10,000 Pierce Universal Nuclease ( 88700; Thermo Fisher Scientific) on ice for 10 min before centrifugating at 4°C for 30 min using a JA20 rotor (334831; Beckman Coulter) at 18,000 rpm. A 10-ml bed of Ni-NTA agarose beads (30250; Qiagen) in a gravity column was equilibrated with lysis buffer. Clear supernatant was supplemented to 20 mM imidazole using 1 M imidazole stock and allowed to flow-through the equilibrated bead bed. The beads were washed with 30 ml high-salt wash (50 mM Tris, pH 8, 2M NaCl, and 20 mM imidazole), followed by 30 ml low-salt wash (50 mM Tris, pH 8, 150 mM NaCl, and 20 mM imidazole). The fusion protein was eluted in 2 × 25 ml fractions of elution buffer (50 mM Tris, pH 8, 150 mM NaCl, 200 mM imidazole, and 0.5 mM TCEP). 200 μl 0.5 M EDTA was added to the eluted protein, and recombinant Ubl-specific protease 1 (ULP) protease was added to a final concentration of 3 μg/ml. The digestion reaction was dialyzed overnight at 4°C in 3.5 L volume of dialysis buffer (for CID–BD: 20 mM Tris, pH 8, 35 mM NaCl, and 0.5 mM TCEP; for CID: 20 mM Tris, pH 8, 150 mM NaCl, and 0.5 mM TCEP) using 10-kDa MWCO SnakeSkin dialysis membrane (68100; Thermo Fisher Scientific). For the CID–BD purification, the sample was further purified after the protease digestion by using a 5-ml Q-HP anion exchange column (17115401; Cytiva) on an AKTA Pure FPLC system, followed by butyl FF hydrophobic interaction chromatography (17519701; Cytiva). The preparation was polished by size exclusion chromatography using a Superdex S75 10/300 gel filtration column (17517401; Cytiva) with gel filtration buffer consisting of 20 mM HEPES, pH 7.5, 100 mM NaCl, and 1 mM DTT. Purified CID–BD was concentrated to >20 mg/ml using 10 kDa MWCO Amicon 15 spin concentrator (UFC901024; Millipore), supplemented with 10% vol/vol glycerol, snap-frozen in liquid nitrogen and stored at −80°C.

For the CID purification, the sample was further purified after the protease digestion reaction by using a tandem 5-ml HisTrap column (17524802; Cytiva) and 5-ml HiTrap Q-HP anion exchange column (17115401; Cytiva) on an AKTA Pure FPLC system. Columns were equilibrated with running buffer (20 mM Tris, pH 8, 150 mM NaCl, and 0.5 mM TCEP) until constant conductance. The sample was loaded using a sample pump at 1.5 ml/min, and fractionation was manually started when A_280nm_ started to increase and continued until A_280nm_ returned to baseline. Fractions from the HisTrap-Q-HP flow-through were concentrated to 500 μl or less final volume using a 10 kDa MWCO Amicon 15 spin concentrator (UFC901024; Millipore) and further purified by size exclusion chromatography using a Superdex S75 10/300 gel filtration column (17517401; Cytiva) with gel filtration buffer (20 mM HEPES, pH 7.5, 150 mM NaCl, and 1 mM DTT). Purified CID at 1–6 mg/ml was supplemented to 10% vol/vol glycerol and aliquoted in 100–300 μl. Protein was flash frozen in liquid nitrogen and stored at −80°C.

### Crystallization and X-ray data collection

TTLL5 CID-RPGR BD crystals were obtained by the sitting drop vapor diffusion method at room temperature using 22 mg/ml of the protein in solution (20 mM HEPES, pH 7.5, and 100 mM NaCl) mixed with an equal volume of crystallization reservoir solution (100 mM sodium citrate, pH 6.4, and 1.625 M Li_2_SO_4_). Crystals grew with symmetry of P 4_1_ 2_1_ 2 with one copy per asymmetric unit (Table 1). Crystals were cryo-protected prior to data collection by quickly soaking in fresh reservoir solution containing 25% glycerol. Crystals were flash frozen by plunging directly into liquid nitrogen. X-ray diffraction data were collected at the Advanced light source beamline 5.0.1 with wavelength of 0.97741 Å. The diffraction data were indexed and integrated using XDS (Kabsch, 2010). The integrated data were then scaled using Scala in the CCP4 program suite (Rigden et al., 2008; Winn et al., 2011). Data collection statistics are reported in Table 1.

The phase solution to the structure was obtained by molecular replacement using PHASER-MR (McCoy et al., 2007) as implemented in *Phenix* (Afonine et al., 2012) using the model for the CID predicted by AlphaFold (Jumper et al., 2021) as the search model. Difference maps revealed unambiguous density for the RPGR BD, which was then manually built into the electron density. Several rounds of iterative model building and refinement were performed using COOT (Emsley and Cowtan, 2004) and Phenix, respectively. All analysis software was accessed through the SBGrid consortium (Herre et al., 2024). The current refined model includes residues 659–811 of CID and residues 1,129–1,152 in the BD. CID residues 770–782 corresponding to the loop between ⍺4 and ⍺ 5 show no interpretable electron density and thus are presumed disordered. The linker between CID and BD is also not resolved. Model statistics are reported in Table 1.

### ITC

ITC was performed using a MicroCal iTC200 calorimeter. CID was thawed and dialyzed overnight at 4°C using Side-A-Lyzer 10 kDa MWCO dialysis cassettes (66383; Thermo Fisher Scientific) into ITC buffer (20 mM HEPES, pH 7.5, and 150 mM NaCl). To ensure chemical equilibrium, the synthesized BD peptide was either reconstituted using the same dialysis buffer used for dialyzing CID or previously reconstituted aliquots were thawed and dialyzed alongside CID using Slide-A-Lyzer mini 2-kDa MWCO dialysis cassettes (69580; Thermo Fisher Scientific). Peptides were synthesized by LifeTein and shipped in lyophilized form. Lyophilized peptides were stored at −80°C until needed. Peptide sequences are RPGR BD WT: SKRLLKNGPSGSKKFWNNVLPHYLELK; RPGR BD F1140A: SKRLLKNGPSGSKKAWNNVLPHYLELK; RPGR BD W1141A: SKRLLKNGPSGSKKFANNVLPHYLELK; RPGR BD L1149A: SKRLLKNGPSGSKKFWNNVLPHYAELK. CID and BD concentrations were determined by 280 nm absorbance measurements using a NanoDrop 2000 spectrophotometer. For measurements with the WT BD peptide, the CID was diluted to 20–50 μM, and the BD peptide concentration was 9–11-fold in excess molar concentration. For measurements with the mutant BD peptides, a CID concentration as high as 200 μM was used, and BD peptide concentration was 9–11 fold in excess molar concentration. ITC cell and syringe were loaded with CID and BD peptide, respectively, according to the manufacturer’s instructions. ITC measurements were performed at 25°C in high feedback mode with one 0.4 μl priming injection, followed by 16 2.45-μl injections with 150-s spacing and stirring at 750 rpm. Binding curves were fitted to a monovalent binding model using NITPIC 1.3.0 and SEDPHAT 15.2b (Zhao et al., 2015). Confidence intervals were calculated using one-dimensional error surface analysis in SEDPHAT.

### AAV plasmid construction and AAV vector packaging

DNA fragments of interest corresponding to the C-terminal tail of RPGR^ORF15^, containing the designed missense mutations and the necessary restriction sites, were synthesized at GENEWIZ. The synthetic fragments were used to swap out the corresponding regions of WT RPGR^ORF15^ to generate RPGR^ORF15^-W1294C and RPGR^ORF15^-L1302A variants. The two variants along with WT RPGR^ORF15^ plasmids were packaged into AAV vectors by triple plasmid transfection into HEK293 cells. AAV vectors were purified by density gradient ultracentrifugation. Vector titers were quantified using TaqMan real-time PCR assays.

### Subretinal AAV injections and immunohistochemistry

Animal care and experimental procedures were carried out in adherence to institutional guidelines and approved protocols. AAV injection was performed as described previously (Sun et al., 2016). Briefly, adult mice were anesthetized, and 1 μl of AAV (3 × 10^12^–8 × 10^12^ viral genomes/ml) was injected subretinally in the nasal quadrant of the retina. Immunohistochemistry was performed as described previously (Sun et al., 2016). Mice were euthanized 12 wk post-AAV injection, and eyes were enucleated. Unfixed eyes were immediately embedded in optimal cutting temperature compound and frozen in dry ice-cooled isopentane. Eyes were sectioned vertically in 10-μm slices. Sections were postfixed in 1% paraformaldehyde and processed as described previously (Hong et al., 2000). Tissue sections were blocked with 5% vol/vol donkey serum in PBS with 0.1% vol/vol Triton X-100 (PBS-Tx) and incubated overnight with primary antibody. After wash with PBS-Tx, slides were stained with fluorescent dye–conjugated secondary antibody and counterstained with DAPI for 2 h. Secondary antibodies used were AlexaFluor 488–conjugated goat-anti-rabbit (A48282; Thermo Fisher Scientific), AlexaFluor 555–conjugated goat-anti-mouse (A48287; Thermo Fisher Scientific), AlexaFluor 488–conjugated donkey-anti-rabbit (A21206; Thermo Fisher Scientific), AlexaFluor 555–conjugated donkey-anti-mouse (A31570; Thermo Fisher Scientific), AlexaFluor 647–conjugated donkey-anti-chicken (703-605-155; Jackson ImmunoResearch), and Dylight549-conjugated donkey-anti-chicken (Jackson ImmunoResearch, discontinued). Confocal microscopy was performed at the NEI Biological Imaging Core Facility. Tissue sections were imaged on a Zeiss LSM 880 AxioObserver scanning confocal microscope with a 40× oil immersion objective lens (EC Plan-Neofluar 40x/1.30 Oil DIC M27) and 405, 488, 561, and 633 nm lasers. The 405- and 633-nm channels utilized photomultiplier tube detectors PMT1 and PMT2, respectively, and 488- and 561-nm channels utilized the gallium arsenide phosphide cathode detector. Objective lens immersion medium was ZEISS Immersol 518F Oil, and tissue sections were imaged at ambient temperature. Micrographs were collected with pixel size of 0.0761 µm/px. Acquisition software is ZEN Black 2.3 SP1, and native image processing software ZEN 3.0 (Black edition) was used to produce maximum intensity projections. Images were processed for figures using ImageJ, Adobe Photoshop (v25) and Adobe Illustrator (v25).

For quantification of RPGR and glutamylation in the connecting cilium, longitudinal traces of the connecting cilium were manually defined in ImageJ, and fluorescence intensity values were extracted. For each cilium, the ratio of GT335 and RPGR signal was calculated at the point of maximum RPGR intensity. Fluorescence intensity was graphed and statistical analysis (unpaired two-tailed *t* test) was performed in Prism 10. For P values <1 × 10^-15^, statistical analysis was repeated in Microsoft Excel to report precise P values. Data distribution was assumed to be normal, but this was not formally tested.

### Glutamylation analysis in the retina by western blot

Glutamylation levels were analyzed by western blot using a procedure similar to the one described previously (Sun et al., 2016). Mouse eyes were stored on ice until neuronal retinas were dissected in a physiological buffer. Retinas were homogenized in RIPA buffer (R0278; Sigma-Aldrich) with complete Protease Inhibitor Cocktail (11836153001; Roche). Tissue debris was removed by centrifugation. Proteins were resolved on a 4–15% SDS-PAGE gel, which was then transferred to a polyvinylidene fluoride membrane. Two replicate gels were run in parallel. Membranes were blocked with EveryBlot blocking buffer (Cat#1201020; Bio-Rad). One membrane was incubated with GT335 overnight at 4°C, while the other with rabbit anti-RPGR. This was due to the lack of suitable secondary antibodies at the time, which we could not order. Membranes were then washed with PBS-0.05% tween-20 (sc-29113; ChemCruz sc-362299), followed by incubation with HRP-conjugated donkey anti-rabbit or anti-mouse IgG (Jackson ImmunoResearch). They were developed by chemiluminescence detection (SuperSignal West Pico, Dura or Femto Chemiluminescent; Thermo Fisher Scientific) and imaged using the ChemiDoc MP Imaging System (Bio-Rad). Mouse monoclonal anti–γ-tubulin (T6557; Sigma-Aldrich) was used as a loading control as can be seen in Fig. 2 B. This method of analysis was used for 2 WT, 1 L1302A, and 1 W1294C, and quantification for this set of experiments is shown with triangles in Fig. 2 C. For 5 WT, 5 L1302A, and 3 W1294C-injected retinas, we used the following experimental design: membranes were incubated with GT335 and rabbit anti-RPGR and then detected on the same membrane using HRP-conjugated donkey anti-mouse and IRDye800-conjugated donkey anti-rabbit (926-32213; LI-COR). RPGR signal was normalized to Glu-tubulin signal, and Glu-RPGR to RPGR ratio was calculated directly. Each circle in Fig. 2 C indicates one retina analyzed. Long exposures were used to identify accurately areas for quantification of faint Glu-RPGR bands (but same exposure settings were used to quantify signal). Band intensities were quantified in ImageJ. The aggregated data from these experiments are shown in Fig. 2 C, with individual data points shown as triangles and circles. Statistical analysis (unpaired two-tailed Welch’s *t* test) was performed in Prism 10. Data distribution was assumed to be normal, but this was not formally tested. Raw, uncropped blots are provided in Source Data.

### Online supplemental material

Fig. S1 shows multiple sequence alignment of vertebrate TTLL5 CID and RPGR BD. Fig. S2 shows comparison of the experimental CID-miniBD X-ray crystal structure with AlphaFold predictions. Fig. S3 shows traced cilia and representative intensity profiles from one biological replicate quantified in Fig. 3 J.

## Supplementary Material

SourceData F2 is the source file for Fig. 2.

## Data Availability

PDB coordinates and structure factors have been deposited with the PDB ID 9PHH. Requests for resources and reagents pertaining to animal lines and AAV-related material will be fulfilled by Tiansen Li (Tiansen.Li@nih.gov). All other requests for reagents will be fulfilled by Antonina Roll-Mecak (Antonina@mail.nih.gov). Source data are included with all figures.
