# Supplementary figures and images for: Insights into retinal disease and non-tubulin glutamylation from a RPGR–TTLL5 complex structure
Source: J Cell Biol. 2026 Apr 3;225(6):e202508020. doi: 10.1083/jcb.202508020 (PMC13052805; doi:10.1083/jcb.202508020)

Membrane 2024-0821\_A (Fig. 2 B)

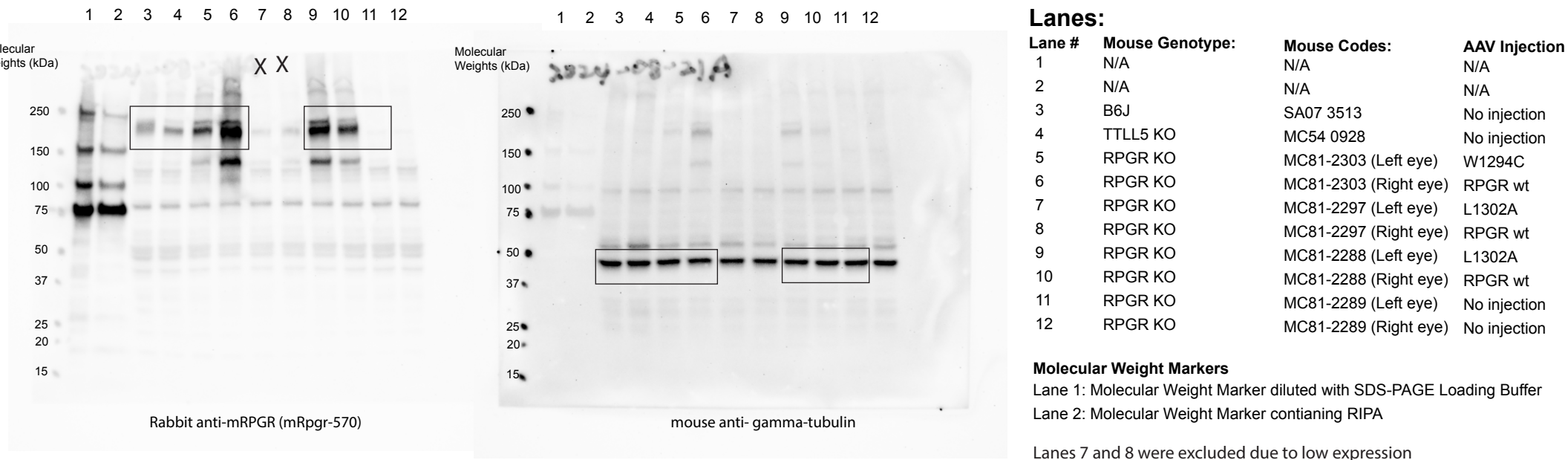

Supplement: SourceData F2 — is the source file for Fig. 2. [file jcb_202508020_sourcedataf2.pdf]
